# Nervous Sympathy

**Published:** 1867-07

**Authors:** 


### NERVOUS SYMPATHY.

On the 12th of June, Mrs. G., of this city, forty-eight years old, and of robust constitution and large breast, consulted me for simple erysipelas, on the back of her left hand. I recommended tinct. of iodine, applied with a soft brush to the part three or four times daily, &c. On the third day she consulted me again, stating that for two days, every time she applied the iodine to the back of her hand, and which invariably produced severe burning, a corresponding effect was produced around each nipple, every whit as intense as if applied to the part. The case is progressing, attended by the same phenomena at each application of the iodine.

It is a fact, well known to the profession, that for several weeks after child birth manipulations of the mamæ will produce "after pains" of greater or less severity, which in some instances recur every time the child sucks, throughout the entire period of lactation. How to account for these things is the difficult part of the subject. It is no explanation to say it is nervous sympathy; of course it is, but what is the *modus operandi?*

